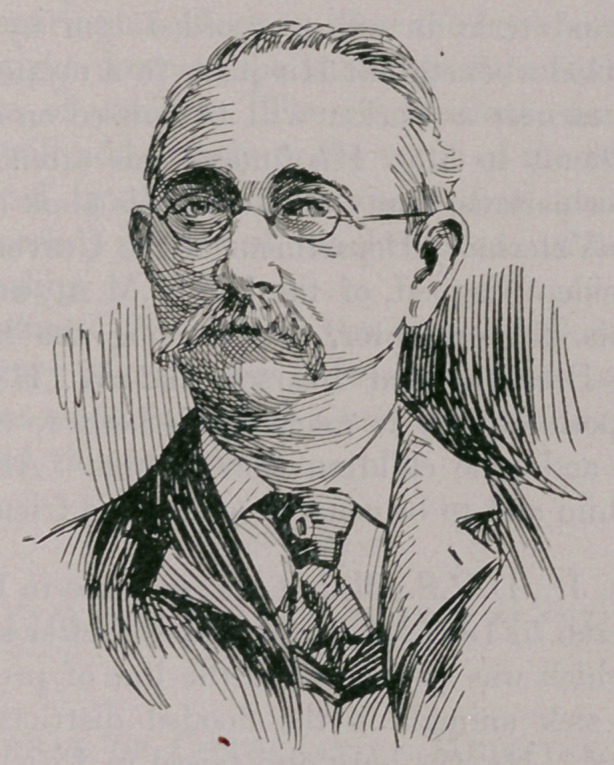# Necrology

**Published:** 1897-04

**Authors:** 


### NECROLOGY.

John R. Hart, V.M.D. At the German Hospital, Philadelphia, March 29, 1897, from acute appendicitis, passed peacefully away one of the best-known and most highly respected veterinarians of our country. Dr. Hart was born in Hammonton, New Jersey, May 9, 1848. Shortly after his parents moved to old Kensington, Philadelphia, where he grew to manhood and spent his entire professional career, and the people learned to prize the noble qualities he possessed. His early life was full of privations and hardships, which he endured with a remarkable degree of courage. He was possessed of a determination to succeed that never faltered in his honorable career. He lived as a citizen among the people who had known him from childhood and as a useful and appreciated practitioner among a large clientage. His early struggles changed in no way the generous, hospitable, and warm-hearted character of the man, and these beautiful traits and disposition were shown forth in his everyday life, and claimed for him a friend with every new acquaintance.

Dr. Hart was graduated from the Veterinary Department of the University of Pennsylvania in the class of 1895 after a period of hard and earnest study, during which he continued his care of a large practice. For thirteen years he was city veterinarian, which position he raised to one of much importance and worth. He was an honored member of the United States Veterinary Medical Association, where on several committees, especially that of Army Legislation, he performed the most earnest and assiduous service; treasurer of the Pennsylvania State Veterinary Medical Association for seven years, where, in addition to caring for the accounts in a most accurate manner, he frequently raised large sums of money for State legislative work, and wielded a strong influence at all times in securing favorable legislation. As President of the Keystone Veterinary Medical Association, an honor conferred upon him in recognition of his valuable services to the profession, he was enjoying a second term in office, accorded him by a unanimous vote. Surely God worketh out His plans in a mysterious way, for so useful and earnest a worker will be missed on every side and his place be difficult to fill. His funeral was attended by a large number of veterinarians, many alumni of his alma mater, and the students of the Veterinary Department of the University of Pennsylvania. President Osgood, of the U.S.V.M.A., and Prof. R. S. Huidekoper, his old preceptor, were among the honorary pall-bearers, while Drs. Leonard Pearson and W. Horace Hoskins were assigned posts of duty in carrying his body to its last resting-place. A wife and nine children survive him. All in all, it was good to know him and to be counted among his friends.

John Doris, Jr., D V.S., died at his residence in Pittsburg, Pa., on Friday, March 5, 1897, of acute pleurisy, after suffering seven days' illness, which was contracted in the line of professional duty while treating sick animals in the flooded districts in the lower parts of the city. He was born and raised in Pittsburg, where he learned the farrier's trade with his father, who was a classical scholar and a well-informed veterinarian and master-horseshoer. Dr. Doris followed his trade for twenty-five years. Then, having decided to study veterinary science, he attended one session at the Ontario Veterinary College, also one session at the Ohio Veterinary College, from which he was graduated and received his degree of D.V.S. April 5, 1892. He was veterinary inspector for three years in the Bureau of Animal Industry, United States Department of Agriculture, and was stationed in Pittsburg. He was a member of the Pennsylvania State Veterinary Medical Association, also of the leading Catholic societies of that section. He was a warm-hearted and generous friend, a good husband, and kind and indulgent father. He was in the fiftieth year of his age, and leaves a widow and three small boys to mourn his demise.

J. W.

Daniel Walsh, V.S., one of Philadelphia's oldest non-graduates, died on March 1st. His practice was largely among the draymen and teamsters along the Delaware River front.

## Figures and Tables

**Figure f1:**